# Dataset of *Escherichia coli* O157: H7 genes enriched in adherence to spinach root tissue

**DOI:** 10.1016/j.dib.2020.105769

**Published:** 2020-05-28

**Authors:** Ashleigh Holmes, Leighton Pritchard, Peter Hedley, Jenny Morris, Sean P. McAteer, David L. Gally, Nicola J. Holden

**Affiliations:** a Cellular and Molecular Sciences, James Hutton Institute, Dundee DD2 5DA, United Kingdom; b The Roslin Institute Division of Infection and Immunity, University of Edinburgh, R(D)SVS, The Roslin Institute Building, Easter Bush EH25 9RG, United Kingdom; c Strathclyde Institute for Pharmacy and Biomedical Sciences, University of Strathclyde, Glasgow G4 0RE, United Kingdom

**Keywords:** Microarray, Bacterial artificial chromosome, Gene enrichment, High-throughput screening, Bacteria-host interactions

## Abstract

A high-throughput positive-selection approach was taken to generate a dataset of Shigatoxigenic *Escherichia coli* (STEC) O157:H7 genes enriched in adherence to plant tissue. The approach generates a differential dataset based on BAC clones enriched in the output, after adherence, compared to the inoculum used as the input. A BAC clone library derived from STEC isolate ‘Sakai’ was used since this isolate is associated with a very large-scale outbreak of human disease from consumption of contaminated fresh produce; white radish sprouts. Spinach was used for the screen since it is associated with STEC outbreaks, and the roots provide a suitable site for bacterial colonisation. Four successive of rounds of Sakai BAC clone selection and amplification were applied for spinach root adherence, in parallel to a non-plant control. Genomic DNA was obtained from a total of 7.17 × 10^8^ cfu/ml of bacteria from the plant treatment and 1.13 × 10^9^ cfu/ml of bacteria from the no-plant control. Relative gene abundance of the output compared to the input pools was obtained using an established *E. coli* DNA microarray chip for STEC. The dataset enables screening for genes enriched under the treatment condition and informs on genes that may play a role in plant-microbe interactions.

Specifications table

**Table:** 

Subject	Microbiology
Specific subject area	Molecular basis to host-microbe interactions
Type of data	Table Microarray genomic DNA datasets
How data were acquired	Microarray: Agilent 8 × 15k *E. coli* gene expression arrays were used with an Agilent G2505B scanner and Agilent FE (AGFE) software v9.5.3.
Data format	Raw: Microarray data was deposited at ArrayExpress, accession numbers of E-MTAB-5923 (plant treatment) and E-MTAB-5924 (control)
Parameters for data collection	There were three considerations for the dataset: (i) the *E. coli* isolate used to generate the BAC library, since STEC (Sakai) is derived from a plant associated outbreak; (ii) the plant host, since leafy greens are a common plant host for STEC outbreaks from fresh produce (iii) the detection system, since microarray is sufficient for enriched gene loci identification
Description of data collection	The data comprises the complete microarray dataset obtained from the enrichment screen, containing genes detected in the plant treatment and control conditions, from input and output pools of BAC clones.
Data source location	The James Hutton Institute Invergowrie, Dundee, Tayside DD2 5DA UK laboratory experiment
Data accessibility	Repository name: ArrayExpress Data identification number: E-MTAB-5923 (treatment); E-MTAB-5924 (control) Direct URL to data: https://www.ebi.ac.uk/arrayexpress/experiments/E-MTAB-5924
Related research article	Ashleigh Holmes^1^, Leighton Pritchard^1^, Peter Hedley^1^, Jenny Morris^1^, Sean P. McAteer^2^, David L. Gally^2^ and Nicola J. Holden^1^, (2020). A role for the plasmid-borne Type II secretion system of *E.* coli O157:H7 (Sakai) in plant-microbe interactions.

## Value of the data

• The data help to determine the molecular basis for how bacteria attach to and interact with plant hosts, and can be directly applied to foodborne pathogens such as STEC, but also to other plant associated bacteria (phytopathogenic, beneficial etc.)
• Those with an interest in plant microbiology, food-borne diseases, environmental microbiology and molecular microbiology will benefit from the dataset.
• These data allow investigation of candidate enriched genes, e.g. in functional analyses for STEC in association with plant or animal hosts; and more widely in identification of orthologous genes in other plant-associated bacteria
• The dataset was derived from a DNA microarray chip, which we have previously shown correlates with deep sequencing-based approaches, and therefore continues to demonstrate the microarray approach as a viable cost-effective tool in a genomic era.

## 1 Data description

STEC are foodborne bacteria that can be transmitted through the food chain on edible produce, which has resulted in large-scale outbreaks of disease. The largest was associated with white radish sprouts from the Sakai district in Japan [5], and leafy greens such as spinach plants are a common source for STEC [7]. Therefore, there is a requirement to identify the molecular basis to how these bacteria colonise plants as alternative hosts. Here, a high-throughput screening approach was adopted for adherence to spinach tissue, using a bacterial artificial chromosome (BAC) library for STEC strain Sakai. The BAC library was hosted in an *E. coli* K-12 strain that does not colonise plant tissue in contrast to the Sakai isolate [4]. The premise to using the BAC library was to obtain a differential dataset of the output from the screen (i.e. adherent) compared to the input applied for the screen (i.e. inoculum). A microarray was used to identify STEC Sakai candidate genes enriched by the adherence screen. We have previously shown that microarray technology is still a viable cost-effective tool in our current genomic era [2], especially for fully characterised genomes, for which the archetypal reference strain STEC Sakai [3] also serves as a relevant plant-associated foodborne pathogen. The microarray contains genomic coverage for both *E. coli* isolate backgrounds (Sakai and K-12-derived strains), thereby allowing a single hybridisation run to obtain all the genomic data required for identification of true positive enriched genes.

The adherence screen used an STEC (Sakai) BAC clone library, comprising 1152 clones, hosted in an *E. coli* laboratory isolate DH10B, derived from *E. coli* K-12. The screen comprised four successive rounds of adherence of the BAC clones to detached spinach roots for two hours (insufficient time for bacterial proliferation), with vigorous washing to remove loosely adherent bacteria. Two amplification steps were included after round 2 and 4 to account for the successive reductions of the number of bacteria recovered from the roots as selectivity increased (Table 1). In the no-plant negative control, the BAC clones were treated similarly and inoculated into medium and suspended in PBS in the absence of spinach root tissue, to account for gene loci in the BAC clone library that may have been enriched during the amplification steps. Genomic DNA was extracted from 7.17 × 10^8^ cfu/ml of bacteria recovered from the plant-treatment and 1.13 × 10^9^ cfu/ml of bacteria from the no-plant control treatment. Gene abundance in pools of BAC clone *g*DNA was quantified on a DNA microarray before (input pools) and after selection (output pools), for both plant-treated and no-plant control conditions to enable differential analyses.

**Table 1:** Numbers of DH10B transformed with Sakai BAC clones (Log_10_ cfu/ml) recovered from the repetitive rounds of adherence to spinach tissue or the control no-plant condition.

Bacterial Counts: stage (total volume)	BAC Pool 1	BAC Pool 2	BAC Pool 3	Control Pool 1	Control Pool 2	Control Pool 3
Input pool (50 ml)	7.375	7.090	7.246	6.804	6.833	6.841
Post Round 1 (2 ml)	NT	5.811	5.828	NT	NT	NT
Post Round 2 (2 ml)	3.398	3.000	3.000	6.892	6.954	6.881
LB amplification (15 ml)	NT	8.477	8.792	8.456	8.425	8.458
MOPS conditioning (15 ml)	NT	9.000	8.986	9.307	9.403	9.255
Post Round 3 (2 ml)	NT	4.000	4.828	NT	NT	NT
Post Round 4 (2 ml)	NT	NT	3.367	9.386	9.599	9.427
Output pool (15 ml)	8.787	8.841	8.928	9.079	9.000	9.079

## 2 Experimental design, materials, and methods

### 2.1 Bacterial strains and media

*E. coli* strains Sakai [3] and DH10B were grown in either lysogeny broth (LB) broth or rich-defined (RD) MOPS medium supplemented with 0.2% glucose, thiamine and essential and non-essential amino acids [6], at 37 °C to obtain bulk cultures, or at 18 °C (indicated) for a plant-relevant temperature. 10 μg/ml Tetracycline (Tet) was used to maintain the BAC clones.

### 2.2 Plant propagation for BAC screen

Spinach (*Spinacia oleracea*) cultivar Amazon seeds (Sutton Seeds, UK) were soaked in sterile distilled water for two hours before being surface sterilised in 2% calcium hypochlorite solution (10 ml) for 10 min. The seeds were washed vigorously six times with sterile distilled water and germinated on distilled water agar (0.5% w/v) in the dark for 3–5 days, at ∼ 22 °C. Seedlings were transplanted into pots containing autoclaved vermiculite and sterile 0.5 x Murashige and Skoog (MS) medium (Sigma Aldrich, USA). Seedlings were grown in a cabinet with a light intensity of 150 μmol m^2^s^−1^ (16 h photoperiod) for a further 21 days at 22 °C.

### 2.3 Bacterial artificial chromosome library screen for adherence to spinach roots

The bacterial artificial chromosome (BAC) library was constructed previously from a partial *Hind*III digest of STEC Sakai genome cloned into the pV41 vector [1]. The BAC library mixture (1152 clones) was cultured in LB at 37 °C for ∼ 18 h. One volume of the cultures was inoculated into 50 vol of RD-MOPS glucose at 18 °C, and incubated with aeration (100 rpm) for 16 h. The BAC colony cultures were combined into three independent 384 clone pools and the cell density adjusted to OD_600_ of 0.02 with sterile PBS to make the inoculum, i.e. the ‘input’ pools. Five spinach roots were harvested and washed in sterile water prior to incubation in 25 ml of inoculum for 2 h, statically at 18 °C (Round 1). Roots were removed and washed three times on a vortex mixer in 15 ml sterile PBS for at least 15 s, full speed. Roots were homogenised with a mortar and pestle in 2 ml PBS and this suspension was added to 15 ml PBS to act as the next inoculum for five new spinach roots (Round 2). After incubation, wash and homogenisation as described before, the homogenate was added to 15 ml LB medium with Tet and incubated at 37 °C, 200 rpm for ∼ 18 h to amplify the enriched clones: the number of bacteria recovered between Round 1 and Round 2 reduced by ∼400-fold (Table 1), therefore an enrichment step after the second round was necessary to increase bacterial numbers for the subsequent rounds of selection. The culture was pelleted, suspended in RD-MOPS glucose and incubated at 18 °C, with aeration (200 rpm) for 16 h to prime the bacteria for the experimental conditions to be tested. Round 3 and 4 of adherence was performed as described above for Rounds 1 and 2, including the LB 18-hour amplification step, resulting in the ‘output’ pools. Hereafter, the bacterial cells from both the input and output pools were harvested for genomic DNA extraction. A control screen was carried out in parallel where the bacteria were not exposed to any spinach roots but had all the same exposure to PBS, temperature and enrichment steps, generating negative control output pools. This accounted for any clones that provided a growth advantage irrespective of plant material, in any of the experimental conditions to be considered in the analysis.

### 2.4 Microarray analysis of DNA

The microarray chip ‘E. coli’ v.2 (Agilent, GEO accession: A-GEOD-13359) contains probes specific for STEC (Sakai) and *E. coli* K-12 isolate MG1655, in addition to accessory genes for two other *E. coli* isolates (STEC EDL933 and uropathogenic *E. coli* CFT073). Genomic DNA was extracted from DH10B containing Sakai BAC clone pools 1–3 recovered from the plant-treatment (7.17 × 10^8^ cfu/ml) and the no-plant control treatment (1.13 × 10^9^ cfu/ml) (Table 1). *g*DNA extraction was performed with a ChargeSwitch Mini Bacteria kit (Invitrogen) exactly as per manufacturer's instructions. Labelling was carried out using a Bioprime Plus Array CGH Genomic labeling System (Invitrogen). Briefly, 100 ng *g*DNA in 11 µl was added to 10 µl 2.5x random primer reaction buffer mix and denatured at boiling for 5 min prior to cooling on ice. To this, 2.5 µl modified 10 × dNTP mix (1.2 mM each of dATP, dGTP, dTTP; 0.6 mM dCTP; 10 mM Tris pH 8.0; 1 mM EDTA), 1 µl of either Cy3 or Cy5 dCTP (1 mM) and 0.5 µl Klenow enzyme was added and incubated for 16 h at 37 °C. Labelled samples for each array were combined (if applicable) and unincorporated dyes removed using Qiaquick PCR Purification Kit (Qiagen) as recommended, eluting twice with 2 × 10 µl EB buffer. Hybridisations and washing were performed as recommended (Agilent Protocol v5.5). DNA was hybridised onto Agilent 8 × 15k *E. coli* gene expression array (Agilent product number G4813A-020,097). Scanning was performed with an Agilent G2505B scanner using default settings and data extracted using Agilent FE (AGFE) software v9.5.3.

## CRediT authorship contribution statement

**Ashleigh Holmes:** Investigation, Formal analysis, Writing - original draft. **Leighton Pritchard:** Investigation, Writing - original draft. **Peter Hedley:** Validation, Data curation. **Jenny Morris:** Investigation. **Sean P. McAteer:** Resources. **David L. Gally:** Resources, Funding acquisition. **Nicola J. Holden:** Conceptualization, Writing - review & editing, Funding acquisition.

## Declaration of Competing Interest

The authors declare that they have no known competing financial interests or personal relationships which have, or could be perceived to have, influenced the work reported in this article.

## Appendix Supplementary materials

Image, application 1
